# How decision analysis can further nanoinformatics

**DOI:** 10.3762/bjnano.6.162

**Published:** 2015-07-22

**Authors:** Matthew E Bates, Sabrina Larkin, Jeffrey M Keisler, Igor Linkov

**Affiliations:** 1 Environmental Laboratory, U.S. Army Engineer Research and Development Center, Concord, MA, USA; 2 Contractor to the Environmental Laboratory, U.S. Army Engineer Research and Development Center, Concord, MA, USA,; 3 Department of Management Science and Information Systems, College of Management, University of Massachusetts Boston, Boston, MA, USA

**Keywords:** decision analysis, nanoinformatics, policy, portfolio analysis, risk assessment, value of information, weight of evidence

## Abstract

The increase in nanomaterial research has resulted in increased nanomaterial data. The next challenge is to meaningfully integrate and interpret these data for better and more efficient decisions. Due to the complex nature of nanomaterials, rapid changes in technology, and disunified testing and data publishing strategies, information regarding material properties is often illusive, uncertain, and/or of varying quality, which limits the ability of researchers and regulatory agencies to process and use the data. The vision of nanoinformatics is to address this problem by identifying the information necessary to support specific decisions (a top-down approach) and collecting and visualizing these relevant data (a bottom-up approach). Current nanoinformatics efforts, however, have yet to efficiently focus data acquisition efforts on the research most relevant for bridging specific nanomaterial data gaps. Collecting unnecessary data and visualizing irrelevant information are expensive activities that overwhelm decision makers. We propose that the decision analytic techniques of multicriteria decision analysis (MCDA), value of information (VOI), weight of evidence (WOE), and portfolio decision analysis (PDA) can bridge the gap from current data collection and visualization efforts to present information relevant to specific decision needs. Decision analytic and Bayesian models could be a natural extension of mechanistic and statistical models for nanoinformatics practitioners to master in solving complex nanotechnology challenges.

## Introduction

Extensive nanomaterial research has yielded an increasing amount of nanomaterial data [1]. The nanomaterial data are currently so vast that it has become difficult to find data relevant to a specific need. However, a formal knowledge infrastructure, inclusive of current nanomaterial data, is essential to future developments in nanomaterial research [2]. Nanoinformatics is defined as (a) “the science and practice of determining which information is relevant to the nanoscale science and engineering community”, and (b) “developing and implementing effective mechanisms for collecting, validating, storing, sharing, analyzing, modeling, and applying that information” [3]. This definition implies the integration of top-down methods for assessing scientific community needs with bottom-up methods for data collection and management [4–5]. Such integration will enhance the reproducibility and distribution of data and the ability to transform the vast nanomaterial data into accessible, integrated information.

Two recent workshops sponsored by the National Nanotechnology Initiative [5] and the National Nanomanufacturing Network [6] were focused on assessing the state of nanomaterial risk management, nanoinformatics, determining gaps in the information and risk management technologies, and evaluating opportunities for improvement. These nanoinformatics workshops highlighted a number of resources that were already using nanoinformatics to aggregate and organize nanomaterial data [6]. The Nanoparticle Information Library (NIL) is a database from the National Institute for Occupational Safety and Health (NIOSH) that aggregates the physical characteristics of nanomaterials for industrial users, researchers, and health professionals to access and share [7]. The NanoHub offers a collaborative workspace for users to share research, identify possible opportunities to work with others, and to learn more about nanotechnology [8]. This includes the GoodNanoGuide, a resource that serves as a best practice exchange for nanomaterials in the workplace [9]. The Nanomaterial Registry archives nanomaterial data according to their properties and environmental and health implications, including their compliance scores [1]. These efforts all focus on developing resources that satisfy the bottom-up part of the nanoinformatics definition presented above. The top-down part, in which the appropriateness of information to a specific need is determined, is not addressed to the same extent in any of the aforementioned efforts. A few existing efforts implement parts of the envisioned top-down strategy but none have bridged the gap to link top-down analytics to the bottom-up data. Some of the closest existing efforts include the various hazard and control banding tools [10], as well as the SUN [11] and LICARA [12] projects of the European Union Seventh Framework Programme. The need for comprehensive top-down approaches was called for after the NNI workshop and decision analytic tools were specifically mentioned as a way of supplementing data intensive visualization methods for the goals of risk management [5,13–14].

For a successful nanoinformatics enterprise, top-down decision analytic tools and bottom-up data management methods need to be integrated. Decision analytic tools are able to bridge the gap between the data needed and the data available to make informed decisions about a new technology. Decision analysis typically formulates models for important decisions in order to identify which alternatives are most desirable given the available information and the preferences of the decision makers, thus incorporating the top-down (decision) perspective. In addition, once decision modeling structures are in place, it is possible to shift attention from selection of alternatives to understanding the data’s support for those alternatives. In other words, decision modeling structures can be used to first synthesize information toward a decision focus and second to identify gaps and delve further in areas of need in order to establish which particular data would be most relevant to the decisions at hand. The ability of decision modeling to identify the relevance of existing data and to distill which areas of research would be most helpful are especially useful when large amounts of data are available and when the data are uncertain and ambiguous.

This paper discusses several decision analytic tools that hold promise for nanoinformatics. We describe the methodology and application of case studies. In particular, we review the use of multicriteria decision analysis (MCDA), value of information (VOI), weight of evidence (WOE), and portfolio decision analysis (PDA) from the perspective of nanoinformatics. We propose that this set of decision analytic methods should be explicitly developed as the next step to advance the nanoinformatics vision of efficiently guiding research and seamlessly identifying and synthesizing available information for decision making.

## Discussion

### Multicriteria decision analysis

Multicriteria decision analysis (MCDA) refers to a set of methods that are employed to rank decision alternatives from most to least preferred. To accomplish this, MCDA allows the user to break down complex problems into more manageable pieces, assess those pieces with respect to the relevant data for each alternative, and reassemble them to present an overall conclusion to decision makers [15]. The process of completing an MCDA can be divided into four steps: (1) identifying the problem, the stakeholders, and the criteria relevant to the decision; (2) extracting weights, thresholds, and other parameters to be inputs in the mathematical model, and assigning measurements for each alternative; (3) executing the model via software; and (4) evaluating the results of the model [16].

MCDA can be applied to nanoinformatics decisions, for example, to help users evaluate and choose a nanomaterial type, formulation, fabrication technique, supplier, coating, or risk management strategy for a new product. From a portfolio of alternatives, MCDA pinpoints those that are most worthy of further consideration based on an aggregated score across all selected evaluation criteria. Most nanomaterial hazard and control banding tools implicitly implement MCDA by using physiochemical property data to relate hazard scores to individual criteria. The criteria are weighted by importance, and the sum of these weighted scores is used to derive an overall hazard score for a nanomaterial. In this way, MCDA-based tools can synthesize data in the context of material development decisions to identify materials with the highest overall hazard scores, typically omitted from use or selected for additional study. The MCDA structure can be used to loosely guide more detailed research and development, because the criteria most in need of further review can be compared in the decision model to find which has the greatest contribution to the overall hazard score [17].

In a case study by Tervonen et al. [18], an MCDA framework was applied for the classification of five nanomaterials: nC_60_, multiwalled carbon nanotubes (MWCNTs), CdSe, silver nanoparticles (Ag NPs), and aluminum nanoparticles (Al NPs). The SMAA-Tri MCDA model was selected as it is well suited for the classification of nanomaterials with uncertain or unavailable physiochemical properties. Five extrinsic characteristics (agglomeration, reactivity, critical functional groups, particle size and contaminant dissociation) and three factors that are dependent on the characteristics listed above and that may influence hazards (bioavailability, bioaccumulation and toxic potential) were used to evaluate the selected nanomaterials [18].

Five alternative risk classifications were proposed for the materials: extreme risk, high risk, medium risk, low risk, and very low risk. The nanomaterials were sorted based on the probability of classification in a particular risk category, given complete information. CdSe was identified as the nanomaterial most likely to receive the highest hazard score, with a 98% chance of being categorized as “high risk.” With these results in mind, the contribution of each criterion to the total score can be evaluated to see which of the eight factors might reasonably benefit from further investigation [18]. This method of determining relevant information with MCDA is a top-down approach. Decision analysis starts with the research objective and ends with decision making. Standard risk assessments, on the other hand, begin with data and end with risk measurements [4]. By starting with the goal of the research, the top-down approach is able to clarify the research needed to achieve the objective and to efficiently make an informed decision.

Beyond this, a series of next steps can be explored to expand the use of MCDA in nanoinformatics. Hazard and control banding tools can be tailored for each funding or regulatory agency’s mission and goals, and additional tools can be developed to meet the needs of other common types of decisions. Furthermore, MCDA capabilities can be integrated into existing nanoinformatics platforms to let users develop their own top-down frameworks, which are linked to the bottom-up data, and to interactively explore evaluations of the best materials for a given design or product. Finally, MCDA can potentially address the need for rapid, real-time screening of nanomaterial hazards and the need for incorporating cost–benefit information alongside environment, health and safety data in a cost–benefit screening.

### Value of information

Value of information (VOI) is a decision analytic concept characterizing the amount a decision maker would pay to acquire additional information that would improve the quality of a decision [19]. As such, it prioritizes research based on its decision relevance, which is the degree to which it is expected to reduce uncertainty regarding the best alternative. Decision relevance is context dependent but vastly more nuanced than approaches that only consider the magnitude of uncertainties in the unweighted and uncontextualized underlying data. Specifically, to calculate the VOI associated with a decision under uncertainty, (i) the best perceived alternative is selected with the benefit of some contemplated information; these outcomes will always be, on average, preferable or at least equal to those of the same decision where (ii) the best perceived alternative is selected in the absence of that information. The expected value of information is the maximum cost which would be spent to get that information while still leaving the decision maker indifferent between (i) and (ii).

The significance of new nanomaterial research and data for a decision maker is often initially unknown. Ideally, further studies would be prioritized such that research plans addressing the greatest amount of uncertainty, or eliminating the uncertainties the decision maker most wants to eliminate, are completed first. The VOI is able to quantify the benefits of this complex bundle of information for a particular decision making situation. In some cases, the VOI also locates a point at which enough information is known, that is, where the marginal returns to additional information diminish to less than the marginal cost of obtaining that information [19].

In a case study from Linkov et al., an MCDA framework evaluates four alternative technologies for single wall carbon nanotube synthesis and a VOI model prioritizes further research [20]. The MCDA process identified pertinent criteria: synthesis cost, material efficiency, energy consumption, life cycle environmental impacts, and risks to human health. A probability distribution of scores for each technology was specified for each criterion via author judgment and the literature. Monte Carlo simulations were used to normalize and aggregate individual criteria distributions into distributions of overall performance using criteria weights associated with preferences of different key stakeholders [20].

After developing result distributions that reflect current uncertainties, the study evaluated research that might best improve decision confidence. Monte Carlo simulations of possible research outcomes (to reduce uncertainty in the input data) and decision outcomes (resulting reduced uncertainty in the distributions of overall scores) were produced for each nanomaterial, showing the likelihood that each nanomaterial would rank first for each stakeholder under different research efforts. This revealed the VOI in terms of increase in the average score of the best alternative selected with the benefit of increasing manufacturing research, health research, both types of research, or neither. The VOI analysis showed that the biggest potential gain in decision confidence in that case would come from health research, which would substantially increase confidence in decisions for both regulators and environmental groups, but not for other stakeholders. In contrast, additional manufacturing research would not substantially improve decision confidence for any of the stakeholders [20]. Applied broadly, this type of analysis can provide a strong basis for identifying and promoting research relevant to future technology development.

A series of next steps can be explored for including VOI in nanoinformatics efforts. Databases can be expanded to include uncertainties for criteria other than hazards (e.g., cost or performance), providing a foundation in the data for the VOI. This is important because research activities that quantify or reduce uncertainty about environmental concerns, material costs, and other cost–benefit parameters are of great value to funding agencies and scientists. Like the suggestion for MCDA technology, VOI algorithms can be imbedded within existing nanoinformatics platforms and tied to the data, putting new capabilities into the hands of the user. Finally, VOI can potentially enable the continuous and immediate classification of uncertainties based on aggregated nanoinformatics data. In this way, the focus could be shifted towards those uncertainties that are relevant to technologies with high potential.

### Weight of evidence

A major challenge in nanoinformatics is how to compare and harmonize the large volume of independently derived, possibly conflicting, and possibly incompatible data into a coherent argument. Weight of evidence (WOE) is a method of integrating and aggregating different and diverse types of evidence to draw a conclusion [21]. The WOE method can be used to fuse information such that discrepancies in data quality and gaps in evidence are considered [21]. WOE was first introduced in the form of a Bayesian model [22] that updates prior beliefs about a hypothesis to form posterior beliefs due to the introduction of new evidence. In this formulation, the Bayes factor is defined as the ratio of prior odds to posterior odds, and the WOE is the natural logarithm of the Bayes factor. More varied qualitative and quantitative applications of the WOE methodology have evolved since then [23].

On the basis of experience with WOE approaches, the National Research Council has recommended a shift towards defensible qualitative and quantitative methods. Quantitative Bayesian approaches and MCDA were both recommended as quantitative supplements and replacements for solely qualitative WOE practices. Thus, the Bayesian approach is able to account for uncertainty and varied sources and types of evidence, while the MCDA approach considers the quality of the evidence and its source as criteria [23]. As in the previous sections, information is first synthesized using the analytical tools, and from this, critical information for decisions or further nanomaterial research is identified.

A case study by Hristozov et al. used a quantitative WOE framework to evaluate the hazards associated with titanium dioxide nanoparticles. Three sets of criteria (physiochemical properties, toxicity, and data quality) were used to evaluate and calculate the hazard scores by means of MCDA. Uncertainties derived from expert judgment were considered in Monte Carlo simulations [24]. As with MCDA, once the WOE hazard score is determined, each contributor to the hazard score can be further reviewed to see which had the largest effect on the score and which might benefit from further research.

A series of next steps can also be explored for including WOE in nanoinformatics efforts. When data is added to nanoinformatics databases, additional quantitative and qualitative metrics (e.g., data statistical significance, precision, applicability, soundness, completeness, uncertainty and variability, degree of review) can be included to contextualize the weight that each data source should carry based on its relevance, quality, resolution, etc. WOE approaches can also be imbedded in nanoinformatics toolsets to help users clarify conflicting and uncertain evidence for early stage nanomaterial evaluations. WOE approaches can be implemented alongside or within hazard and control banding tools to allow differentiation between input data. In the future, continuous and immediate application of a standardized WOE approach with nanoinformatics data could provide a real-time and more accurate initial summary of nanomaterial hazards or other conclusions that can be drawn from the body of knowledge [24].

### Portfolio decision analysis

Portfolio decision analysis (PDA) is similar in aim to the tools discussed earlier, but with one major distinction: instead of choosing one option from a set of choices, a subset of items (a portfolio) is selected [25]. The MCDA, VOI, and WOE methods are all appropriate for use with either single choice decision analysis or portfolio decision analysis. Once a series of possible portfolios has been evaluated, the portfolios with the highest score at any given budget or level of resource availability can be further investigated. The nanomaterials that contribute most to the portfolio score will be identified, along with the qualities shared among the high scoring nanomaterials.

Bates et al. applied PDA to sets of nanomaterial hazard research efforts, in order to prioritize research portfolios at the national level. This PDA was an extension of a VOI approach evaluating multiple research topics for three emerging nanomaterials: multiwalled carbon nanotubes, silver nanoparticles, and titanium dioxide nanoparticles [26]. First, a preliminary screening tool (CB Nanotool 2.0 [17], an MCDA-based approach) was used to assign distributions of hazard scores for each characteristic of a chosen nanomaterial. These scores were summed across properties to assign a distribution of overall hazard scores for each material. Based on these total scores, the materials were probabilistically classified as high risk, moderate risk, and low risk.

From there, the VOI model estimated the improvement in hazard-identification accuracy for each unique research effort. Each research effort was assumed to reduce the uncertainty associated with a single parameter for a single nanomaterial. Research portfolios for each nanomaterial were defined as sets of research efforts addressing parameters for that material. Monte Carlo simulations were used to estimate the expected benefit of each research effort and portfolio, with the assumption that research undertaken on a material property would reveal a true hazard score prior to the decision, and otherwise, that score would only become known after material classification. For each realization of the simulation, the correct score and classification of the material are assumed to be the score and classification identified when all parameter values are known. The proportion of realizations for which a research portfolio is expected to lead to the correct classification and the degree to which it produces hazard scores matching the correct hazard scores can be tabulated. By comparing this performance to that of a baseline portfolio in which no research is done, it is possible to determine the average increase in value for each research portfolio. These calculations are properly performed at the portfolio level because the potential for any given effort to affect a material’s classification and significantly reduce hazard uncertainty depends on the state of knowledge of other parameters for the material [26].

To better reflect the national decisions that are typical of funding agencies, the portfolios of research efforts were also aggregated across materials. Plotting each aggregated portfolio’s increase in performance against its difficulty or cost revealed an efficient set of most desirable portfolios (those with a value higher than any others of similar cost) [26]. It is then simple to inspect any of these types of portfolios and observe what research on which nanomaterials and properties might be most attractive at different levels of overall investment.

A series of next steps can also be explored for including PDA in nanoinformatics efforts. Funding agencies, research institutions, corporations, and individual research teams can use nanoinformatics data with PDA techniques to help prioritize future research efforts. PDA algorithms can be tailored to work more seamlessly with existing and future MCDA, VOI, and WOE tools supporting decisions in nanotechnology. Finally, as with the other tools, PDA algorithms can be added to nanoinformatics tool sets to put greater top-down analytical power in the hands of the end user.

## Conclusion

Recent discussions from the Nanotechnology Knowledge Infrastructure have heralded the creation of a communication portal for the various nanotechnology databases and tools. The tremendous amount of data that would be available via that portal would necessitate not only the bottom-up accumulation, sorting, and visualization of data, but the top-down identification of decision-relevant information. The four tools described here can accomplish both facets of that goal, and overall, provide capability to expand the reach of current nanoinformatics tools.

Part of this expansion should be accomplished through use of expert elicitations, which are often featured in decision analysis to supplement and connect hard data to the decision while leaving a transparent record of the way in which this connection is made. In the context of nanoinformatics, properly implemented human judgments can help users navigate and incorporate available information resources. Each of the applications described herein uses such judgments. The weights on criteria for a given stakeholder are nearly always subjectively assigned (although they use techniques that are transparent, maximize logical consistency, and minimize psychological biases). While some uncertainties involving the outcome of repetitive processes can be readily characterized on the basis of statistical data, it may be impossible or inadequate to do so in situations involving new or ambiguous factors. It is a philosophical point emphasized in decision analysis that in making choices, it is rational for decision makers to act consistently with what is implied by their beliefs in conjunction with the information they have.

The use and implementation of these decision analytic techniques are not without challenges [27]. These include involving the right experts and stakeholders so that results will be credible, guarding against motivational and other biases in elicitation and dissemination [28], and communication of results in a way that they will be known, understood and trusted by the people who can use them [29]. In addition, the academic decision analysis community is often focused on the creation of new tools, and is less interested in their immediate application. Open advocacy and networking from the community could better relay the benefits of these approaches and techniques.

Thus expanded from information retrieval to decision support, nanoinformatics has the potential to improve the characterization of nanomaterials, the reproducibility of nanomaterial research, and the accessibility of data. Currently, nearly all nanoinformatics efforts are working from a bottom-up perspective to create databases and archives and to organize all of the available data instead of employing a top-down decision approach to identify relevant data. Without the incorporation of both top-down and bottom-up concepts, the full definition and scope of the nanoinformatics vision may not be realized. A range of decision analytic techniques, starting with MCDA, VOI, WOE, and PDA, as described here, can help to sort through and organize the vast nanomaterial data to inform both current choices and the prioritization of future nanomaterial research. These techniques focus the attention of researchers and policy makers toward what is most relevant to their decisions and provide consistent and transparent frameworks for integrating that information. In the future, we expect that both decision analytic techniques and Bayesian models will be used as extensions of standard mechanistic and statistical models to leverage and advance developments in nanoinformatics [21].
